# The presence of β_2_-adrenoceptors sensitizes α_2A_-adrenoceptors to desensitization after chronic epinephrine treatment

**DOI:** 10.1186/1471-2210-7-16

**Published:** 2007-12-20

**Authors:** Tasneem Bawa-Khalfe, Ghazi F Altememi, Chitra D Mandyam, Lindsay A Schwarz, Douglas C Eikenburg, Kelly M Standifer

**Affiliations:** 1 Tasneem Bawa-Khalfe, Research Center for Cardiovascular Diseases, Brown Foundation Institute of Molecular Medicine for the Prevention of Human Diseases, The University of Texas-Houston Health Science Center, Houston, TX, USA; 2 Chitra D. Mandyam, Committee on the Neurobiology of Addictive Disorders, The Scripps Research Institute, La Jolla, CA, USA; 3 Kelly M. Standifer, Ph.D., Dept. Pharmaceutical Sciences, 1110 N. Stonewall, Suite 329, University of Oklahoma Health Sciences Center, Oklahoma City, OK, USA; 4 Department of Pharmacological and Pharmaceutical Sciences, University of Houston, Houston, TX, USA

## Abstract

**Background:**

In addition to the regulation of blood pressure, α_2_- and β-adrenoceptor (AR) subtypes play an important role in the modulation of noradrenergic neurotransmission in the human CNS and PNS. Several studies suggest that the α_2_-AR responsiveness in cells and tissues after chronic epinephrine (EPI) or norepinephrine (NE) exposure may vary, depending on the β-AR activity present there. Recently, we reported that in BE(2)-C human neuroblastoma cells (endogenously expressing α_2A_- and β_2_-AR), chronic EPI treatment (300 nM) produced a dramatic β-adrenoceptor-dependent desensitization of the α_2A_-AR response. The aim of this study is to determine if stable addition of a β_2_-AR to a second neuroblastoma cell line (SH-SY5Y), that normally expresses only α_2A_-ARs that are not sensitive to 300 nM EPI exposure, would suddenly render α_2A_-ARs in that cell line sensitive to treatment with the same EPI concentration.

**Methods:**

These studies employed RT-PCR, receptor binding and inhibition of cAMP accumulation to confirm α_2_-AR subtype expression. Stable clones of SH-SY5Y cells transfected to stably express functional β_2_-ARs (SHβ_2_AR4) were selected to compare sensitivity of α_2_-AR to EPI in the presence or absence of β_2_-ARs.

**Results:**

A series of molecular, biochemical and pharmacological studies indicated that the difference between the cell lines could not be attributed to α_2_-AR heterogeneity. We now report that after transfection of functional β_2_-AR into SH-SY5Y cells (SHβ_2_AR4), chronic treatment with modest levels of EPI desensitizes the α_2A_-AR. This effect results from a β_2_-AR dependent down-regulation of native α_2A_-ARs by EPI accompanied by enhanced translocation of GRK2 and GRK3 to the membrane (required for GRK-mediated phosphorylation of agonist-occupied receptors).

**Conclusion:**

This study further supports the hypothesis that the presence of the β-AR renders the α_2A_-AR more susceptible to desensitization with physiological levels of EPI.

## Background

Studying changes in α_2_-adrenoceptor (AR) signaling is important for understanding the development and/or manifestation for several CNS (cerebral ischemia, pain, depression) and PNS disorders (hypertension and cardiac dysfunction). Under physiological conditions, norepinephrine and epinephrine (NE and EPI, respectively) activate the α_2_-AR along with other members of the AR family, which also includes α_1_- and β-ARs. The α_2_- and β-ARs are often co-expressed on the same cell surface. Upon activation by NE and EPI, the independent signals initiated by the α_2_- and β-ARs often converge to regulate specific physiological endpoints such as insulin release [1], maintenance of uterine smooth muscle tone [2], and noradrenergic transmission in the CNS and PNS [3,4]. The α_2_- and β-ARs regulate many of these physiological mechanisms by mediating opposing actions on adenylyl cyclase; α_2_-AR inhibits while β-AR stimulates the adenylyl cyclase pathway.

Continuous exposure to catecholamines leads to a declining receptor response, a phenomenon called desensitization. The process of desensitization generally includes receptor phosphorylation, internalization, and down-regulation. Unlike other members of the AR family, the α_2A_-AR subtype does not readily down-regulate. Since this subtype is the dominant α_2_-AR in the CNS and mediates the "classical effects" of α_2_-ARs which include hypotension, sedation, and antinociception [5,6], numerous studies have focused on the regulatory mechanisms of the α_2A_-AR. In cultured cell lines expressing either native α_2A_-AR [7] or recombinantly over-expressed α_2A_-AR [8,9], supra-physiological concentrations of EPI (100 μM) and NE (30 μM) were required to produce long-term α_2A_-AR desensitization. The waning α_2A_-AR signal is attributed primarily to down-regulation of the receptor and/or phosphorylation of the agonist occupied receptor by G-protein coupled receptor kinases (GRK), specifically GRK2 and GRK3 [10,11]. Previous studies suggest that either of these two α_2A_-AR desensitization mechanisms require supra-physiological (μM) concentrations of agonist [10,12-14].

However, our recent studies in the BE(2)-C human neuroblastoma cell line suggest that when β-ARs are present on the same cells lower, more physiologically relevant, concentrations of EPI (300 nM) are able to desensitize the α_2A_-AR following chronic (24 hr) treatment [15]. In the absence of β-ARs, α_2A_-AR desensitization occurs only with supra-physiological concentrations of EPI, if it occurs at all [15]. Concurrent activation of the β-AR and α_2A_-AR also prompts down-regulation of cell surface α_2A_-ARs while specifically up-regulating the expression of GRK3 within BE(2)-C cells [15]. Enhanced GRK3 expression plays a prominent role, as it is required for both β-AR-dependent α_2A_-AR desensitization and down-regulation [15,16]. Recently we reported similar findings for the α_2B_-AR subtype in mouse neuroblastoma cells [17-19].

Since both α_2_- and β-ARs are often co-localized and share the same endogenous ligands, it is reasonable that the α_2A_-AR response is regulated differently in the presence and absence of the β-AR. Indeed, evidence suggests that the α_2_-AR responsiveness in cells and tissues after chronic EPI or NE vary, depending on the β-AR activity present there [2,15,20-23]. The aim of the present study is to compare α_2A_-AR responsiveness after chronic EPI and NE treatment in non-β-AR expressing (wild-type SH-SY5Y, wt) human neuronal cells to α_2A_-AR responsiveness in SH-SY5Y cells that have been stably transfected to express β_2_-AR (SHβ_2_AR4). In doing so, we hope to determine whether co-expression of the two ARs intrinsically produced this differential α_2A_-AR regulation and whether enhanced expression of GRK3 is required for this regulation.

## Results

### Characterization of the model system and establishment of the SHβ_2_AR4 cell line

Our first goal was to find a second model system that was similar to the BE(2)-C human neuroblastoma cell line (expressing modest levels of α_2A_-AR), but that didn't express β-ARs. Kazmi and Mishra previously identified the SH-SY5Y cell line as expressing two α_2_-AR binding sites [24], while Parsley *et al.*[25] reported that it expressed a single AR subtype, α_2C_, based upon functional and molecular studies. Since receptor expression varies depending on differentiation state and passage number, it was necessary to determine which α_2_-AR subtypes were expressed in our population of SH-SY5Y cells, using a combination of binding, functional, and molecular approaches.

SH-SY5Y cells expressed α_2_-AR levels slightly greater than the level detectable in BE(2)-C cells (B_max_: SH-SY5Y, 67.6 ± 8.2 ; BE(2)-C, 40.8 ± 7.0 fmol/mg protein). According to nonlinear and linear regression analysis of saturation binding, the data best fit a single-site model in SH-SY5Y cells, as observed previously in BE(2)-C cells. Rauwolscine and yohimbine competed for specific [^3^H]rauwolscine binding to SH-SY5Y cell membranes with higher affinity than prazosin, the α_2B/C_-selective antagonist (Table 1; [24]). Apparent K_*i *_values of agonists and antagonists against [^3^H]rauwolscine binding were determined for comparison with previously reported values in cells natively expressing α_2A_-, α_2B_, or α_2C_-ARs (HT29 and BE(2)-C, NG108-15, OK; [15,26,27]) or cell lines expressing cloned α_2_C10, α_2_C2, and α_2_C4 [28]. Values obtained from binding studies in SH-SY5Y cells correlated only to values from BE(2)-C cells and showed the greatest similarity with those derived from native and cloned α_2A_-AR-containing cell membranes (Table 2). These results are consistent with binding of [^3^H]rauwolscine to an α_2A_-AR in SH-SY5Y cells.

**Table 1:** Pharmacological characteristics of adrenoceptors in SH-SH5Y and SHβ_2_AR4 cells.

	**SH-SY5Y**		**SHβ_2_AR4**
**Agonist:**	**log(K_*i*_)**	**log(EC_50_)**	**log(EC_50_)**

EPI	-7.38 ± .04	-8.83 ± .06	-8.22 ± 0.21
UK 14,304	-7.38 ± .12	-7.22 ± .36	-7.72 ± 0.77
Oxymetazoline (OXY)	-8.85^a^	-8.35 ± .47	N.D.
Isoproterenol	N.A.	N.A.	-7.02 ± 0.28

**Antagonist:**	**log(K_*i*_)**	**K_*i *_Ratio with OXY**	

Rauwolscine	-8.82 ± .15	1.07	N.A
Yohimbine	-8.56 ± .17	1.95	N.A
Prasozin	-6.98^a^	74.4	N.A

Binding inhibition and cAMP accumulation studies were performed as described in *Methods*. The values of the apparent affinity constants Log(K_*i*_) for each competitor were derived from their IC_50 _values (n = 3–9) using the equation of Cheng and Prusoff [40]. The Log(EC_50_) values (concentration of the drug that produces 50% of the maximal inhibitory/stimulatory effect of that drug) were calculated by nonlinear regression analysis of the agonist concentration-response curves (n = 3–9) of each agonist. ^a^Values from Kazmi and Mishra [24]. N.D., not determined; N.A., not applicable.

**Table 2:** Correlation of SH-SY5Y cell α_2_-AR *p*K_*i *_values with those of native and cloned α_2_-AR subtypes.

**Comparison**	**Reference**	**# of Values Compared**	**Correlation Coefficient**	**Slope**	***p *value**
*v. *HT29	21,22	6	0.93	1.48 ± 0.41	0.07
*v. *NG108-15	21	6	0.13	0.17 ± 0.91	0.87
*v. *OK	21,22	6	0.62	0.99 ± 0.73	0.27
*v. *α_2_C10	23	6	0.80	1.04 ± 0.45	0.10
*v. *α_2_C2	23	6	0.40	0.48 ± 0.65	0.50
*v. *α_2_C4	23	6	0.70	0.94 ± 0.52	0.16
*v. *BE(2)-C	6	6	0.98*	1.38 ± 0.18	**0.01**

Correlation coefficient values (r) were generated by comparing *p*K_*i *_values from Table 1 with previously published values for one-site models using Pearson correlation analysis (GraphPad Prism). The slope of the linear regression line is also included. Correlations were considered significant (*) if *p *≤ 0.05.

**Figure 1:**
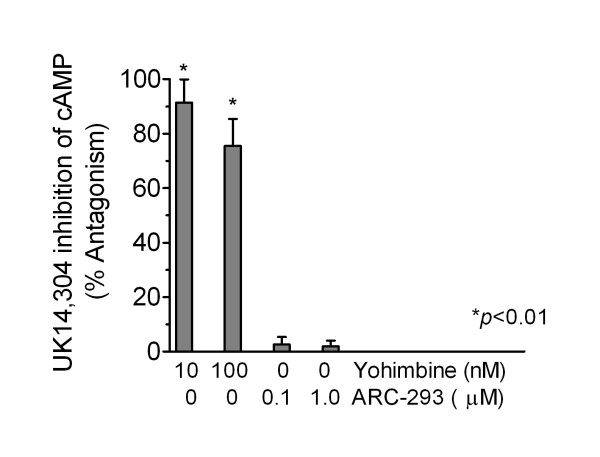
**Reversal of the inhibitory effect of UK 14,304 on forskolin-stimulated cAMP accumulation**. Yohimbine significantly antagonized the ability of UK14,304 (30 nM) to inhibit cAMP accumulation using an unpaired Student's *t*-test (GraphPad Prism, San Diego, CA), while the α_2B/C_-selective antagonist, ARC-239, had no effect. The results represent the mean ± S.E. of 2–9 experiments, performed in duplicate.

Functional studies were performed by measuring the ability of various α_2_-AR agonists to inhibit forskolin (10 μM)-stimulated cAMP accumulation in intact cells. All α_2_-AR agonists inhibited forskolin-stimulated cAMP accumulation in a concentration-dependent manner; no stimulation of cAMP accumulation was noted in the absence of forskolin. Inhibition of cAMP accumulation by the α_2_-AR agonist UK14,304 (30 nM; Fig. 1) was completely reversed by 10 nM yohimbine, whereas the α_2B/C_-selective antagonist ARC-239, at a concentration over 30-fold higher than that of the agonist, failed to reverse the actions of UK14,304. Thus, both binding and functional data support the classification of the α_2_-AR subtype in this neuroblastoma cell line as α_2A_.

**Figure 2:**
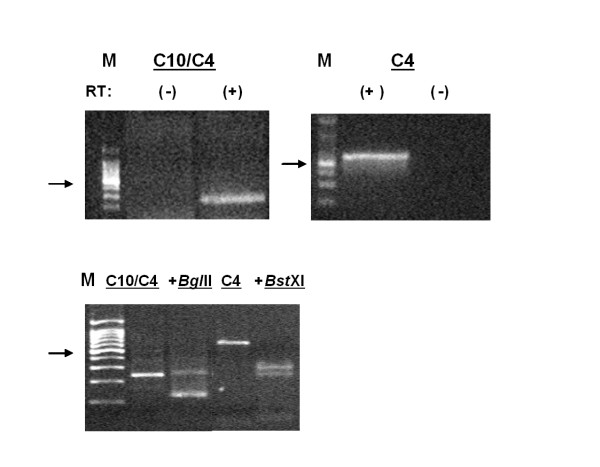
**RT-PCR Products obtained from SH-SY5Y RNA using α_2_-AR subtype selective primers**. RT-PCR experiments were performed as described in "Methods" using primer pairs recognizing α_2_C10/C4 (corresponding to α_2A _and α_2C_) and α_2_C4 (corresponding to α_2C_) gene products (Table 3). The reactions amplified fragments of the expected size from each set of primers. α_2_C10/C4 primers amplified 233 bp products from SH-SY5Y mRNA that were sensitive to digestion by *Bgl*II (specific for the α_2A _product). Restriction digestion with *Bst*XI of the 630 bp product of α 2C4 primer amplification gave three fragments of 271, 225 and 78 bp. All reactions were performed in the presence (+) or absence (-) of reverse transcriptase (RT) to rule out the possibility of DNA contamination. Lane M designates the 100 bp ladder; the 500 bp fragment is indicated by an arrow in each panel.

Since Parsley *et al.*[25] were unable to detect α_2A_-AR RNA by performing RT-PCR with total RNA extract, we optimized our chances for detecting α_2A_-AR RNA by generating RT-PCR products from SH-SY5Y mRNA using primer pairs selective for individual α_2_-AR subtypes (Table 3; [29,30]) or a primer pair that recognizes two α_2_-AR receptor subtypes distinguished by their restriction nuclease digestion products (Table 3; [30]). RT-PCR with α_2_C10/C4 primers gave a 233 bp product specific for α_2A_- and α_2C_-ARs; restriction digestion of this fragment with *Bgl*II, that would specifically cleave α_2A_-AR, resulted in two fragments of 117 bp and thereby established expression of α_2A_-AR mRNA in SH-SY5Y cells. RT-PCR with α_2_C4 primers gave a 630 bp fragment, which was successfully digested by *Bst*XI to produce three fragments of 271, 225, and 78 bp, consistent with the presence of an α_2C_-AR gene product (Fig. 2). RT-PCR products were neither noted in samples lacking reverse transcriptase (-), nor were they produced with primers selective for α_2_C2 (α_2B_-AR; data not shown). While SH-SY5Y cells express mRNA for both α_2A_- and α_2C_-ARs, it appears that the predominant functional α_2_-AR in our cell line is the α_2A_-AR.

**Table 3:** Molecular characteristics of α_2_-AR RT-PCR products

**PCR Product**	**Primer:**	**Receptor**	**Expected size (bp)**	**Restriction Enzyme**	**Digestion Products (bp)**
α_2A/C_**-**AR	α_2_C10/C4	α_2A_	233	*Bgl*II	117 (2)
		α_2C_	233	S*ac*I	153, 80
α_2C_**-**AR	α_2_C4	α_2C_	630	*Bst*XI	271,225,78

Since these cells appear to express α_2A_-ARs with properties similar to those in BE(2)-C cells [15] but lack a β-AR, pcDNA 3.0 plasmid vector containing the human β_2_-AR gene was transfected into SH-SY5Y cells. Colonies of stable transfectants were selected and maintained by their resistance to G418 (600 μg/mL) and subsequently clonal populations of β_2_-AR-expressing SH-SY5Y cells (SHβ_2_AR) were screened for β-AR expression using [^3^H]CGP-12177 for binding studies as described in Methods. Since BE(2)-C cells express very low levels of β_2_-AR (B_max_: 18.5 ± 6.2 fmol/mg protein), the SHβ_2_AR4 cell line that expressed 14.78 ± 4.19 fmol/mg protein of the β_2_-AR was selected for the subsequent studies. To ensure that the β-ARs were functional, the ability of isoproteranol (ISO) to stimulate cAMP accumulation was assessed (Table 1). The α_2A_-AR responses were also tested in this new cell line to confirm that α_2A_-AR function had not been altered by the expression of the β_2_-AR (Table 1).

### Chronic 300 nM EPI exposure induces α_2A_-AR desensitization only in SH-SY5Y cells transfected with functional β-AR

**Figure 3:**
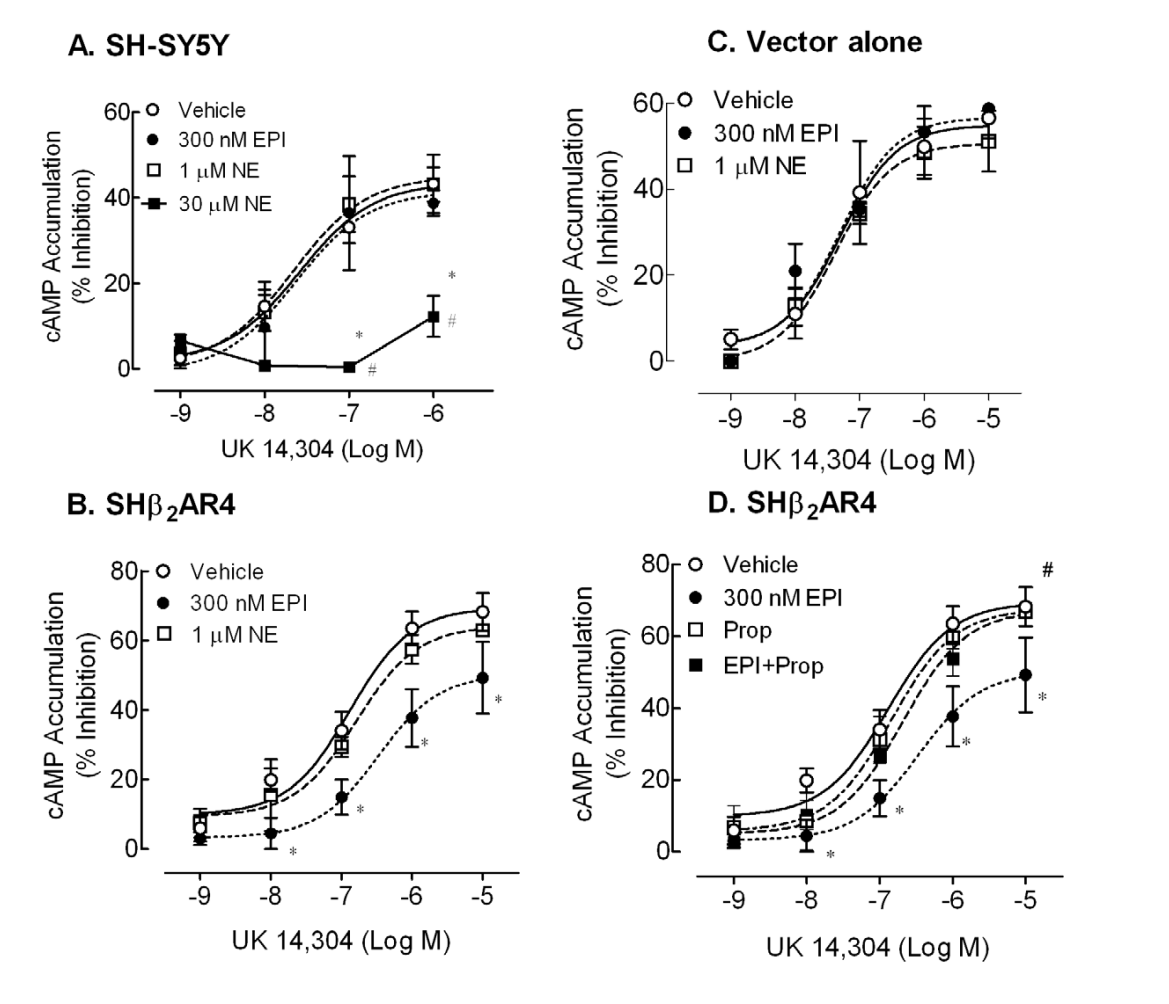
**Pretreatment with a modest concentration of EPI produces α_2A_-AR desensitization in SH-SY5Y cells only when the β_2_-AR is present**. Wild-type SH-SY5Y cells (**A**), cells expressing recombinant β_2_-AR (SHβ_2_AR4, **B **and **D**), or SH-SY5Y cells expressing the vector alone (**C**) were pretreated 16–24 hr with any or all of the following: EPI (300 nM), NE (1 μM or 30 μM), EPI + Prop (30 nM), Prop (30 nM) alone or vehicle (0.1 mM ascorbate). Following pretreatment, the ability of UK14,304 to inhibit forskolin-stimulated cAMP accumulation was evaluated. **A**) Neither chronic EPI nor 1 μM NE pretreatments were sufficient to alter the α_2A_-AR signal (n = 6) in native SH-SY5Y cells. The α_2A_-AR signal in these cells desensitized only when exposed to higher agonist concentrations (30 μM NE, n = 3; 100 μM EPI, n = 3, **data not shown**). **B**) Unlike native SH-SY5Y cells, pretreatment with 300 nM EPI is sufficient to desensitize the α_2A_-AR signal in SHβ_2_AR4 cells (n = 6; p < 0.05). NE (1 μM), acting predominantly at α_2A_-AR with little affinity for the β_2_-AR, does not produce α_2A_-AR desensitization. **C**) In SH-SY5Y cells transfected with the vector alone, neither EPI nor NE pretreatments altered α_2A_-AR signal (n = 4).**D**) Addition of propranolol (30 nM) prevents EPI-induced α_2A_-AR desensitization, suggesting a β_2_-AR-dependent process (# p < 0.05 as compared to EPI treatment).

To determine whether the presence of the β-AR influences α_2A_-AR signaling, the ability UK14,304 to inhibit forskolin-stimulated cAMP accumulation was evaluated after wildtype (wt) and SHβ_2_AR4 cells were exposed to vehicle or the indicated concentration of agonist for 16–24 hr. Wt SH-SY5Y cells (Fig 3A) require a 30-fold higher concentration of NE (30 μM) to desensitize the α_2A_-AR signal than SHβ_2_AR4 cells (1 μM; Fig 3B). Both the potency (-Log EC_50 _(M): 5.2 ± 0.1) and efficacy (I_max _(%): 17.0 ± 1.6; *P *< 0.05 Fig. 3A) of UK14,304 were reduced by 30 μM NE compared to vehicle treatment in wt cells (-7.6 ± 0.2 M and 43.2 ± 6.8%); modest concentrations of NE (1 μM) and EPI (300 nM) are insufficient to alter the α_2A_-AR signal in the wt SH-SY5Y cell line. In contrast, chronic treatment of the β-AR-expressing SHβ_2_AR4 cells with 300 nM EPI desensitized the α_2A_-AR signal causing loss of UK14,304 potency (-Log EC_50 _(M): Vehicle 6.9 ± 0.2; EPI 6.3 ± 0.2) and efficacy (I_max _(%): Vehicle 68.2 ± 5.4; EPI 49.3 ± 10.4; p < 0.05; Fig. 3B). Unlike EPI, which co-activates both ARs, NE, at the concentrations employed activates only α_2A_-ARs and does not alter α_2A_-AR signaling. We concluded that the difference in α_2_-AR signaling following EPI treatment between the transfected and wt SH-SY5Y was attributable to the presence of functional β_2_-ARs, respectively. To ensure that the vector was not responsible for the observed difference between the wt and SHβ_2_AR4 cells, similar experiments were conducted in SH-SY5Y cells transfected with the vector alone (minus the β_2_-AR gene). These vector only-expressing clones responded to EPI (300 nM) and NE (1 μM) pretreatments as the parent SH-SY5Y cells did (Fig. 3C).

To validate the importance of the β_2_-AR in the desensitization of the α_2_-AR signal, we included the β-AR selective antagonist propranolol (30 nM) with the chronic 300 nM EPI treatment. Addition of propranolol blocks EPI-induced α_2A_-AR desensitization resulting in UK14,304 concentration-response curves indistinguishable from control (-Log EC_50 _(M) for EPI + Prop 6.7 ± 0.1; I_max _(%) for EPI + Prop 67.9 ± 0.4; p < 0.05; Fig. 3D). Propranolol treatment alone did not alter UK14,304 potency or efficacy.

### β_2_-AR signal is desensitized following exposure to 300 nM EPI

**Figure 4:**
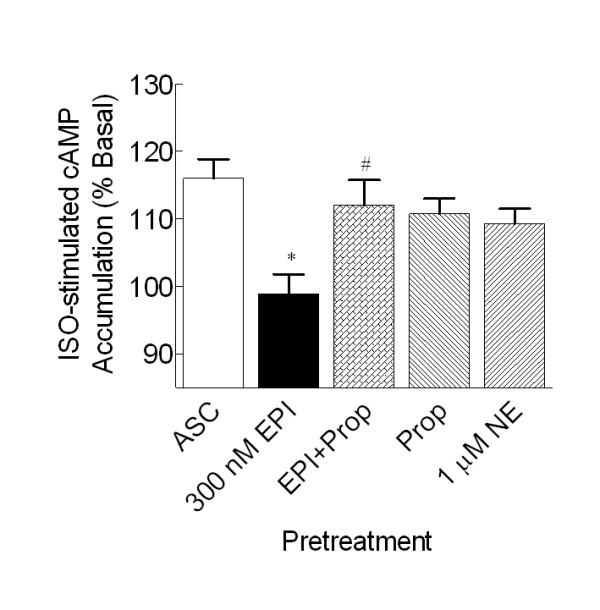
**Chronic EPI, but not NE, treatment desensitizes the β_2_-AR signal in SHβ_2_AR4 cells**. SHβ_2_AR4 cells were treated for 16–24 hr with the vehicle (ascorbate, 1 μM), EPI (300 nM), NE (1 μM), EPI + Prop (30 nM), or Prop (30 nM) alone. Intact cells were assessed for ISO-stimulated (250 nM) cAMP accumulation. Chronic 300 nM EPI (n = 6; *P < 0.05), but not 1 μM NE (n = 3), pretreatment desensitized the β-AR response to ISO compared to the corresponding vehicle-treated control. The β-AR antagonist propranolol blocked EPI-induced β_2_-AR desensitization. Data represent mean ± S.E. of at least 3 independent determinations; comparisons were made by ANOVA with Dunnett's post-hoc test.

To ensure that the β_2_-AR is functioning properly following catecholamine treatment, we evaluated the ability of ISO to stimulate cAMP accumulation over basal in SHβ_2_AR4 cells. The β_2_-AR signal is desensitized following chronic EPI but not NE treatment, consistent with the fact that NE has a low affinity for the β_2_-AR. Inclusion of propranolol (30 nM) inhibited EPI-induced β_2_-AR desensitization (p < 0.05; Fig. 4), but had no effect in the absence of EPI.

### Chronic EPI-induces down-regulation of the α_2A_-AR in SHβ_2_AR4, but not wt SH-SY5Y cells

**Figure 5:**
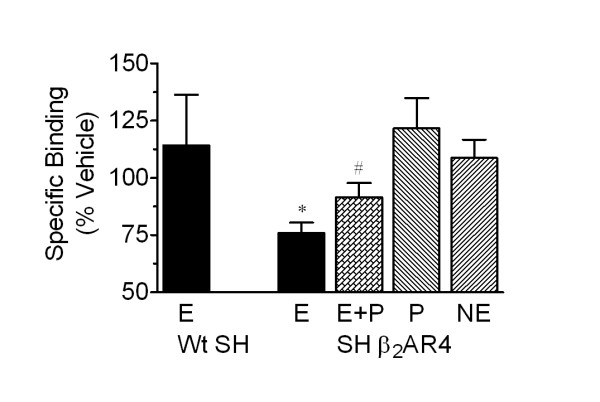
**Chronic 300 nM EPI down-regulates α_2A_-AR in β_2_-AR-transfected, but not native, SH-SY5Y cells**. Wt SH-SY5Y or SHβ_2_AR4 cells were incubated for 16–24 hr with vehicle (ascorbate, 0.1 mM), 1 μM NE, 300 nM EPI, EPI + Propranolol (30 nM), or 30 nM Propranolol alone. Cell membrane homogenates were generated as described in *Methods*. Specific binding (8084 ± 609 cpm/mg protein in vehicle-treated cells) was calculated by subtracting the binding of a single concentration of radioligand (2 nM) in the presence of phentolamine (10 μM) from the binding in its absence. Unlike in native cells, chronic EPI treatment reduced α_2A_-AR levels as compared to vehicle (*p < 0.05); inclusion of propranolol blocked the EPI-induced α_2A_-AR down-regulation (^#^p < 0.05 as compared to EPI treatment) in SHβ_2_AR4 cells. Data represent mean ± S.E., n = 2–4; comparisons were made by ANOVA with Tukey's post-hoc test.

Our study in BE(2)-C cells suggests that β_2_-AR-induced α_2A_-AR desensitization following long-term EPI exposure is due in part to down-regulation of the α_2_-ARs. To determine if the same mechanism is responsible for the EPI-induced α_2A_-AR desensitization in SHβ_2_AR4 cells, changes in α_2A_-AR expression following catecholamine treatment were evaluated. Specific binding was measured with a single concentration of radioligand. We, and others, have shown that this is sufficient for accurate assessment of changes in receptor number for the α_2A_-AR [9,15]. Chronic exposure of SHβ_2_AR4 cells to 300 nM EPI down-regulates the α_2A_-ARs by 20% (p < 0.05; Fig. 5). The α_2A_-AR down-regulation in this cell line, as in BE(2)-C cells, requires β_2_-AR co-activation since loss of α_2A_-ARs is prevented when 30 nM propranolol is included with EPI. Down-regulation of the α_2A_-AR is not observed following chronic activation of α_2A_-AR alone by 1 μM NE. Further, 300 nM EPI does not alter the expression of α_2A_-AR in wt SH-SY5Y cells as compared to vehicle-treated cells (% of vehicle: 88.6 ± 25.9; n = 2) consistent with a lack of α_2A_-AR desensitization. Hence, it can be concluded that chronic EPI treatment induces a loss of α_2A_-AR response via β_2_-AR-dependent down-regulation of α_2A_-ARs in SHβ_2_AR4, but not in wt SH-SY5Y cells.

### Chronic EPI exposure does not alter GRK2 or GRK3 levels in whole cells but instead enhances GRK2 and GRK3 expression at the membrane in SHβ_2_AR4 cells

We previously established that EPI-induced α_2A_-AR desensitization and down-regulation in BE(2)-C cells is mediated via β_2_-AR-dependent GRK3 up-regulation [15]. Therefore, GRK3 levels in whole cell SHβ_2_AR4 lysates were evaluated following 24 hr catecholamine treatments. Chronic EPI exposure altered neither GRK3 nor GRK2 levels in the transfected SH-SY5Y cell line (Table 4). Therefore, unlike results in BE(2)-C cells, increases in whole cell GRK3 levels do not contribute to the modest α_2A_-AR desensitization or down-regulation observed in the SHβ_2_AR4 cells.

**Table 4:** Total GRK levels are unaltered in SHβ_2_AR4 cells with catecholamine treatment.

	Catecholamine Treatment*			
	
	**EPI**	**EPI+P**	**Prop**	**NE**
GRK3	94 ± 8 (7)	84 ± 15 (7)	80 ± 20 (7)	84 ± 20 (5)
**GRK2**	101 ± 10 (7)	97 ± 16 (7)	105 ± 14 (7)	83 ± 23 (4)

SHβ_2_AR4 cells were treated with vehicle (0.1 mM ascorbate), EPI (300 nM), propranolol, EPI + propranolol, or 1 μM NE for 24 hr. Approximately 25 μg of whole cell lysate from each treatment group was resolved by SDS-PAGE through a 10% gel. Immunoreactive bands were normalized to the GAPDH loading control and the GRK/GAPDH ratio was calculated.* Data represent % of expression levels noted in vehicle-treated cells (mean ± s.e.m.); number of independent determinations is given in parentheses following the values.

**Figure 6:**
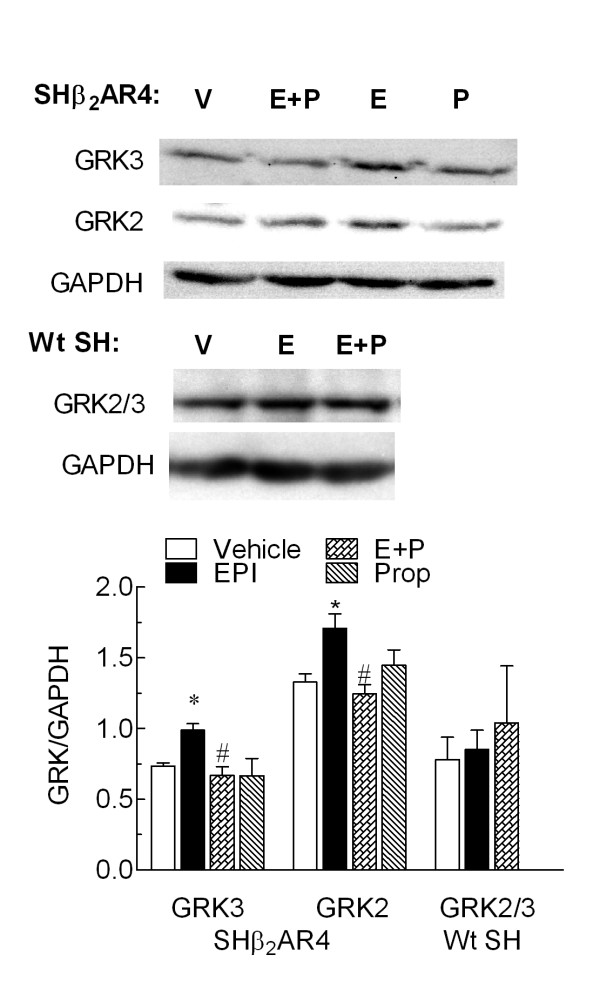
**Chronic 300 nM EPI enhances expression of GRK3 and GRK2 at the membrane of SHβ_2_AR4 cells via β_2_-AR-dependent mechanism**. Wildtype SH-SY5Y (Wt SH) and SHβ_2_AR4 cells were subjected to catecholamine treatment in the presence or absence of 30 nM propranolol. Isolation of the membrane fraction and immunoblotting for GRK2 and GRK3 was conducted as described in *Methods*. EPI exposure significantly increased the level of GRK3 and GRK2 expressed in the membrane fractions from SHβ_2_AR4 cells compared to vehicle-treated controls (*P < 0.05; n = 3). Inclusion of propranolol (P) with EPI treatment prevented the increased translocation of both GRK isoforms (^#^*P *< 0.01 as compared to EPI treatment), while propranolol treatment alone was without effect. In contrast, EPI failed to increase mobilization of GRK to the plasma membrane of wt SH cells (n = 4–7). Data represent mean ± S.E.; comparisons were made by ANOVA with Tukey's post-hoc test.

Although GRK3 levels in whole cell lysates remain unaltered in SHβ_2_AR4 cells, it is not known whether GRK3 recruitment to the membrane is regulated via chronic EPI treatment in that cell line. Since GRK2 and GRK3 have been shown to regulate α_2A_-AR signaling [10], we wanted to determine whether the membrane recruitment of either GRK isoform was changed following chronic EPI exposure in SHβ_2_AR4 cells. GRK2 and GRK3 are cytosolic proteins that anchor to the membrane via interaction with free Gβγ subunits; thus both kinases translocate from the cytosol to the membrane to regulate receptor signaling upon activation. Taking this characteristic of GRK2 and GRK3 into account, the levels of both kinases in membrane fractions following chronic EPI exposure were evaluated. SHβ_2_AR4 cells exhibit an increase in membrane-associated GRK2 and GRK3 with 24 hr EPI treatment compared to vehicle (*P *< 0.05; Fig. 6). In SHβ_2_AR4 cells, the same propranolol concentration (30 nM) that inhibited EPI-induced α_2A_-AR desensitization and down-regulation also attenuated EPI-induced increase in GRK2 and GRK3 content in the membrane fraction (*P *< 0.05; Fig. 6). In contrast, no increased translocation of GRKs by EPI treatment was observed in wt SH-SY5Y cells that do not express β_2_-ARs. Therefore, this increased GRK2 and GRK3 translocation to the membrane following prolonged EPI treatment in SHβ_2_AR4 cells is β_2_-AR dependent.

## Discussion

The major finding of the present study is the confirmation (using a different approach) that sensitivity of α_2A_-AR to desensitization following exposure to relatively low levels of epinephrine is significantly increased in cells expressing both α_2A_- and β_2_-AR. The first evidence for this was recently reported in a human neuronal cell line endogenously expressing α_2A_- and β_2_-ARs. Alpha_2A_- and β-ARs in BE(2)-C cells desensitized after chronic EPI (300 nM), but not NE (1 μM), treatment [15]. Interestingly, the α_2A_-AR responsiveness in SH-SY5Y cells (an alternative human neuroblastoma cell line that does not express β-ARs) is not desensitized after chronic treatment with 300 nM EPI or 1 μM NE (Fig. 3).

Obviously, the difference in α_2_-AR sensitivity to lower concentrations of EPI could be due to several factors, including differences in the α_2_-AR subtypes expressed in each cell line. Since it is difficult to demonstrate with great certainty what α_2_-AR subtypes are present in a given cell or tissue by biochemical or pharmacological means only, we took a molecular approach to ascertain which subtypes might potentially be expressed based on the presence of mRNA encoding each subtype. SH-SY5Y cells contained mRNA for α_2A_- and α_2C_-ARs (Fig. 2). As we noted and as reported by others [25], no evidence for α_2B _mRNA was found. This was further confirmed by Northern blot analysis (data not shown). Initially, total RNA isolated from SH-SY5Y cells did not produce the α_2A_-AR PCR products using the antisense primer selective for α_2A_-AR previously described [30]. Instead α_2A_-AR RT-PCR product was obtained only with poly(A) mRNA. However, employing poly(A)-enriched mRNA in the RT-PCR did not yield an α_2B_-AR RT-PCR product. Parsley et al. [25]) identified only α_2C_-AR mRNA using total RNA isolated from SH-SY5Y cells; this observation may reflect the limitation associated with the α_2A_-AR primers used for RT-PCR of total RNA, similar to what we encountered.

The rank order binding affinity of the various agonists and antagonists tested is in agreement with that previously reported in cells expressing recombinant [3,28,31] or native α_2A_-ARs [26,32]. When we compared apparent pK_*i *_values for various α_2_-AR agonists and antagonists against binding to [^3^H]rauwolscine in SH-SY5Y membrane homogenates with previously reported values, we saw a correlation only with those cells that expressed α_2A_-ARs (Table 2). Another means of distinguishing between various α_2_-AR subtypes involves comparing the prazosin/oxymetazoline (OXY) or OXY/yohimbine affinity ratios (Table 1; [4]). Prazosin/OXY (74.4) and OXY/yohimbine (1.95) ratios were within the range reported for native and recombinant α_2A_-ARs, and differ by at least 10-fold from values reported for α_2C_-AR (*from *[4]). The agonist potency series in SH-SY5Y cells also most closely parallels that reported for the α_2A _[26-28,32]. The inhibitory effect of α_2_-AR agonists on cAMP production in SH-SY5Y cells is readily reversed in a concentration-dependent fashion by the antagonist yohimbine (Fig. 1); the failure of the selective α_2B/C _antagonist ARC-239 to antagonize UK 14,304 is consistent with activation of α_2A_-ARs in SH-SY5Y cells. Therefore, our results strongly support the designation of the functional α_2_-AR in SH-SY5Y cells as α_2A_.

The present study supports our previous findings that pretreatment with a modest EPI concentration readily desensitizes the α_2A_-AR signal in the presence, but not in the absence, of the β_2_-AR. This conclusion is based on several results. First, in wt SH-SY5Y cells (no β_2_-AR), the α_2A_-AR signal is not desensitized following 24 hr treatment with modest concentrations of EPI or NE (300 nM and 1 μM, respectively). Instead wt cells required chronic exposure to supra-physiological concentrations of catecholamines (30 μM NE and 100 μM EPI; data not shown) for desensitization of the α_2A_-AR signal; supporting the fact that α_2A_-ARs do not desensitize and/or down-regulate readily in response to low to moderate levels of EPI. Second, 300 nM EPI induces α_2A_-AR desensitization only in SHβ_2_AR4 cells which express functional β_2_-AR. Finally, EPI-generated waning of the α_2A_-AR response is not observed in transfected cells expressing the pcDNA plasmid vector minus the β_2_-AR gene. This observation suggests that introduction of the β_2_-AR, and not the vector, is responsible for the difference in the α_2A_-AR signal between wt and SHβ_2_AR4 cells exposed chronically to modest EPI concentrations.

As previously observed in BE(2)-C cells [15], desensitization of α_2A_-AR signal with 24 hr EPI exposure is due, in part, to down-regulation of the receptor in SHβ_2_AR4 cells. Chronic co-activation of both α_2A_- and β_2_-AR is required for desensitization and down-regulation of the α_2A_-AR in SHβ_2_AR4 cells as indicated by the following results. First, 300 nM EPI, but not 1 μM NE, produces α_2A_-AR desensitization and down-regulation in the recombinant cell line. Lands et al. [33] established that EPI has equal affinity for α_2A_- and β_2_-AR while NE has a higher affinity for the α_2_-AR than β_2_-AR; therefore, EPI activates both α_2A_- and β_2_-ARs simultaneously while NE activates the α_2A_-AR alone. It is evident that the modest EPI concentration readily activates the β_2_-AR since chronic pretreatment with 300 nM EPI, but not 1 μM NE, desensitized the β_2_-AR response. Second, the inclusion of the β_2_-AR blocker propranolol prevented EPI-induced α_2A_-AR desensitization and down-regulation in β_2_-AR-transfected SH-SY5Y cells. This propranolol concentration (30 nM) is sufficient to prevent EPI activation of β_2_-AR as indicated by the inhibition of EPI-induced β_2_-AR desensitization.

Although chronic EPI treatment desensitized and down-regulated α_2A_-AR in both BE(2)-C and SHβ_2_AR4 cells, several differences were observed. First, a more profound loss of efficacy is observed following 24 hr EPI exposure in BE(2)-C cells as compared to SHβ_2_AR4 cells. The maximal inhibition of forskolin-stimulated cAMP accumulation by UK14,340 was reduced 54% in BE(2)-C, but only 27% in SHβ_2_AR4, cells following EPI treatment (Fig. 3). The greater down-regulation of α_2A_-ARs observed in BE(2)-C versus SHβ_2_AR4 cells most likely accounts for the greater change in efficacy: in SHβ_2_AR4, chronic EPI treatment produces a 20% loss of α_2A_-ARs while in BE(2)-C cells, there is a 60% α_2A_-AR down-regulation (Fig. 5). This more profound α_2A_-AR desensitization and down-regulation observed in BE(2)-C is mediated via the up-regulation of GRK3 [15]. The lack of GRK3 up-regulation in the SHβ_2_AR4 cells is the second major difference between the two cell lines. At present, it is unknown what prompts GRK3 up-regulation in BE(2)-C cells but not in the SHβ_2_AR4 cells. However, we have observed that ERK1/2 activation is required for this induction of GRK3 following chronic exposure of BE(2)-C cells to EPI [16]. Moreover, while α_2A_-ARs do not readily activate this pathway in neuronal cells, and β-AR activation by ISO can activate ERK1/2 at high concentrations, we have observed that ERK1/2 activation by EPI at concentrations that up-regulate GRK3 appears to require the simultaneous activation of both α_2A_- and β_2_-ARs. Conversely, the inability of transfected β-ARs to prompt ERK1/2 activation in SH-SY5Y cells could explain the lack of GRK3 up-regulation in SHβ_2_AR4 cells.

Even though total GRK3 levels are unaltered, GRKs play a role in β_2_-AR-regulated α_2A_-AR signaling in SHβ_2_AR4 cells as indicated by several results. First chronic EPI treatment enhances localization of GRK2 and GRK3 to the membrane. As indicated previously, translocation of these two cytosolic kinases to the membrane is required for phosphorylation and subsequent desensitization of its receptor substrate, which in this study is the α_2A_-AR. Second, addition of propranolol attenuated EPI-mediated translocation of both GRK isoforms. This same propranolol concentration also inhibited α_2A_-AR desensitization and down-regulation as discussed above. Therefore, β_2_-AR co-activation with α_2A_-AR is required for enhanced GRK2 and GRK3 translocation to the membrane and subsequent α_2A_-AR desensitization and down-regulation.

Translocation of both GRK2 and GRK3 to the membrane following chronic EPI treatment in SHβ_2_AR4 cells differs from the selective translocation of GRK3 (but not GRK2) observed in BE(2)-C cells following the same treatment (unpublished observations). The selective GRK3 up-regulation in BE(2)-C cells could account for the enhanced GRK3 levels at the membrane in these cells since in a previous study increase in total GRK2 levels promoted increased GRK2 expression at the membrane [34]. It is unknown at present why chronic EPI treatment translocates both GRK2 and GRK3 in SHβ_2_AR4 cells, and not in BE(2)-C cells. A possible explanation for the difference in the GRK isoform translocation between the two cell lines is differences in the β subunit expressed and/or released upon EPI exposure. GRK2 and GRK3 require the βγ subunit of the G proteins to anchor to the membrane but GRK2 and GRK3 exhibit distinct binding preferences for individual β subunits [35]. The β_3 _isoform preferentially binds GRK3 but not GRK2, whereas β_1 _and β_2 _bind equally to both GRK3 and GRK2 [35,36].

## Conclusion

Based on results obtained in this series of experiments, we conclude that exposure to modest EPI concentrations readily desensitizes and down-regulates α_2A_-ARs in the presence, but not in the absence, of a functional β-AR. The β-AR-dependent down-regulation of α_2A_-ARs is modulated via GRKs. In BE(2)-C cells, chronic co-activation of β- and α_2A_-AR prompts enhanced expression of GRK3, but not GRK2, in whole cells [15] and membrane fractions. In contrast, EPI pretreatment of SH-SY5Y cells transfected with functional β_2_-ARs does not increase either GRK3 or GRK2 expression per se, but does increase translocation of GRK2 and GRK3 to the plasma membrane. Like α_2A_-AR desensitization and down-regulation, this translocation of GRK2 and GRK3 in SHβ_2_AR4 cells is β-AR-dependent and thus presents an alternate mechanism for the regulation of the α_2A_-ARs by β-ARs.

## Methods

### Materials

The following drugs were purchased or obtained from the indicated sources: (-) epinephrine (EPI), (±)norepinephrine (NE), sodium ascorbate, UK14,304 (Sigma-Aldrich, St. Louis, MO.); cell culture media (Gibco, Grand Island, NY); fetal bovine serum (Atlanta Biologicals, Norcross, GA); and antibiotics (Mediatech, Inc., Herndon, VA). GRK2 (C-15) and GRK3 (C-14) primary antibodies and horseradish peroxidase-conjugated secondary antibody (Santa Cruz Biotechnology, Inc., Santa Cruz, CA); anti-glyceraldehyde-3-phosphate dehydrogenase (GADPH, Research Diagnostics, Inc., Flanders, NJ).

### Cell culture

SH-SY5Y (passages 37–55) human neuroblastoma cells (Dr. Robert A. Ross, Fordham University, Bronx, NY) were maintained in a humidified atmosphere (6% CO_2_:94% air) in a 1:1 mixture of Eagle's minimum essential medium with non-essential amino acids and Ham's F-12 that contains 10% fetal bovine serum, 100 U/ml penicillin G and 0.1 mg/ml streptomycin sulfate. Plates of cells greater than 60% confluence were used throughout the study.

### Transfection

Plasmid cDNA with the human β_2_-AR gene (provided by Dr. Brian Knoll; University of Houston, Houston, TX) or vector alone was stably transfected into SH-SY5Y cells with the fuGENE 6 Transfecting Reagent (Roche). Ten positive clones were isolated by their resistance to 800 μg/mL of G418 and maintained in media containing 600 μg/mL of G418. SHβ_2_AR4 was selected for use in all experiments because it expressed similar levels of β_2_-ARs as that expressed natively in BE(2)-C cells; SHβ_2_AR4 expressed 14.78 ± 4.19 fmol/mg protein while BE(2)-C express 18.5 ± 6.2 fmol/mg protein [15]. This β_2_-AR level remained consistent to passage 12 in SHβ_2_AR4 cells. After passage 12, SHβ_2_ARs neither expressed β_2_-ARs nor maintained resistance to G418, suggesting that the cells no longer expressed the transfected plasmid.

### RNA isolation and RT-PCR

Total RNA was isolated from several different passages of freshly harvested SH-SY5Y cells by the guanidinium isothiocyanate/phenol-chloroform extraction method [37]. Total RNA concentrations were determined by UV spectroscopy; integrity of each isolate was determined by electrophoresis through a 1% agarose gel in the presence of 0.01 M sodium phosphate buffer. Poly(A) mRNA was isolated using a Dynabead oligo(dT)_25 _Kit (Dynal, Oslo, Norway) and was used for RT-PCR reactions. Each RT reaction (20 μL) contained 5–10 μg total or poly(A) RNA preincubated with 5 ng/μL oligo(dT)_12–18_, for 10 min at 70°C. The reaction mixture contained 80 μM each of deoxynucleotides (dATP, dCTP, dGTP and dTTP), RT buffer (50 mM Tris-HCl, pH 8.3, 75 mM KCl, 3 mM MgCl_2_), and 5 mM dithiothreitol, and was preincubated for 2 min at 42°C before the addition of Moloney Murine Leukemia Virus reverse transcriptase (200 U/μl) for 60 min at 42°C; a 5 min incubation at 95°C terminated the reactions.

ODNs [29,30] corresponded to sequences for the various human α_2_-AR (α_2A _antisense: 5'-AGA CGA GCT CTC CTC CAG GT-3'; sense: 5'-AAA CCT CTT CCT GGT GTC TC-3'), α_2A/2C_-(antisense: 5'-GTG CGC TTC AGG TTG TAC TC-3'; sense: 5'-AAA CCT CTT CCT GGT GTC TC-3'), or α_2C_-AR (antisense: 5'-CGT TTT CGG TAG TCG GGG AC-3'; sense: 5'-GTG GTG ATC GCC GTG CTG AC-3'). The contents of each RT reaction tube were diluted to a final volume of 50 μL with 10% DMSO, 80 μM each of dATP, dCTP, dGTP and dTTP, 8 μM each of the appropriate sense/antisense primer pair, 1.5 mM MgCl_2_, and magnesium free buffer [containing 10 mM Tris-HCl (pH 9.0), 0.1% Triton X-100, 50 mM KCl] in sterile distilled water. Reaction mixtures were overlayed with mineral oil and subjected to a hot start for 5 min at 95°C. DNA polymerase (2.5 U *taq*, 5 U/μl, Promega, Madison, WI) was added to each reaction tube after the hot start, and the tubes were subjected to a PCR reaction of 30 cycles in a thermal cycler (MJ Research Inc., Watertown, MA) for 1 min at 94°C, 1.5 min at 55°C, and 2 min at 72°C with a final elongation step at 72°C for 7 min. Reaction products were separated by electrophoresis through 2% agarose gels and visualized by ethidium bromide staining. PCR products were isolated from the gel using a DNA extraction kit (Amicon Inc., Bedford, MA). Identity of the purified PCR products was confirmed by their susceptibility to digestion with restriction enzymes specific for each reaction product (see Table 1; [30]).

### cAMP accumulation

To determine the effects of α_2_-AR agonists on forskolin-induced cAMP accumulation, intact cells were incubated for 5 minutes at 37°C in HBSS buffer (in mM): NaCl (137), KCl (5), Na_2_HPO_4 _(0.6), KH_2_PO_4 _(0.4), NaHCO_3 _(4), D-glucose (6), MgCl_2 _(0.5), MgSO_4 _(0.4) and CaCl_2 _(1), containing the phosphodiesterase inhibitor IBMX (0.5 mM). In some experiments, antagonists also were included in this step. To prohibit oxidation, sodium ascorbate (0.11 mM) was included when assaying catecholamines. Upon addition of forskolin (10 μM) and agonist, assay tubes were incubated for an additional 10 min at 37°C. Removing the tubes to a boiling water bath for 5 min terminated the assay. All assays were performed in duplicate in a total volume of 0.5 ml. After boiling, samples were centrifuged for 5 min at 14000 × *g*, and cAMP levels from the supernatant fractions were determined in a [^3^H]cAMP (0.8 pmol) binding assay as previously described [38]. β-AR-mediated stimulation of cAMP accumulation was performed in the same manner except that forskolin was not included in the assay mixture. Forskolin (10 μM) stimulated cAMP accumulation to 587 ± 88 pmol/mg protein (n = 46), 15-fold over basal levels (40.5 ± 2 pmol/mg protein).

### Receptor binding

#### Preparation of cell membranes

Cells were homogenized in 20 volumes of Tris-HCl buffer (50 mM, pH 7.4) containing NaCl (100 mM), Na_2 _EDTA (1 mM) and PMSF (0.1 mM), and the membranes sedimented by centrifugation for 30 minutes at 34000 × *g *at 4°C. Pellets were resuspended in 0.32 M sucrose, and aliquots of the membrane fractions were stored frozen (-80°C) until use.

#### Saturation experiments

The level of α_2_-ARs in SH-SY5Y cell membranes (0.5 mg/ml) was determined with various concentrations of [^3^H]rauwolscine (60–80 Ci/mmol, 0.3 – 12 nM) in a total volume of 1–2 ml in potassium phosphate buffer (50 mM, pH 7.4) containing MgSO_4 _(5 mM) at 37°C for 45 min. Thereafter, 2 ml Tris-HCl (5 mM, pH 7.4, 4°C) was added to the homogenate to terminate the binding reaction and the contents of the tubes was filtered over #32 glass fiber filter strips (Schleicher & Schuell, Keene, NH) using a PHD cell harvester (Cambridge Technology, Cambridge, MA). The reaction tubes and the filter strips were rinsed twice with a further 2–3 ml of buffer. Levels of radioactivity were determined by scintillation spectroscopy in a Beckman LS6000 liquid scintillation counter. All assays were performed in triplicate, and specific binding was determined by subtracting the binding in the presence of yohimbine or phentolamine (10 μM; nonspecific) from the binding in its absence.

Previously we have shown that agonist treatments do not alter the K_d _of the ligand for the α_2_-AR [15]. Therefore, levels of α_2_-ARs in SHβ_2_AR4 cell membranes (0.1 – 0.2 mg/mL) were determined using a single concentration (2 nM) of either [^3^H]rauwolscine or [^3^H]RX821002 following catecholamine treatment.

β_2_-AR binding was performed with [^3^H]CGP-12177. For saturation studies, cell membranes (0.5 mg/mL) were incubated with [^3^H]CGP-12177 (0.2 to 40 nM) in Tris-HCl buffer (50 mM, pH 7.5) containing MgCl_2 _(0.5 mM) at 37°C for 30 min. Specific binding was determined by subtracting the binding in the presence and absence of propranolol (1 μM).

#### Competition experiments

Cell membrane fractions were incubated as described above except that the concentration of [^3^H]rauwolscine was fixed (1 nM), and various (4–9) concentrations of unlabeled drugs were included.

#### Immunoblotting

Membrane proteins were separated from cytosolic proteins by centrifugation, were resolved by SDS-PAGE through 10% gels and relative levels of GRK2 and GRK3 determined by immunoblotting as described previously [15]. Briefly, proteins were transferred to PVDF membrane, blocked with 5% nonfat dried milk in PBS containing 0.1% Tween (PBS/T) and incubated overnight at 4°C with dilutions of a rabbit polyclonal antibody directed against GRK2 (1:1000), GRK3 (1:1000), or both GRK2 and GRK3 (GRK2/3; 1:1000; wt SH-SY5Y). Blots were subjected to 4 washes before incubating for 60 min at room temperature with a goat anti-rabbit horseradish peroxidase-conjugated secondary antibody (1:2000) in PBS/T. Immunoreactive bands were visualized by enhanced chemiluminescence (Amersham Corp., Arlington Heights, IL or Santa Cruz Biotechnology, Inc., Santa Cruz, CA). The intensity of each immunoreactive band was determined using a Nucleovision Imaging Workstation (Nucleotech Corp., San Carlos, CA), and normalized to the GAPDH loading control (1:5000).

### Protein determination

Bovine serum albumin was used as a standard in the determination of protein levels in intact cells and cell membranes as described [39].

### Data analysis

K_d_, B_max_, IC_50 _and LogEC_50 _values were determined by nonlinear regression analysis using GraphPad Prism (GraphPad Software ). The K_i _values were calculated according to the Cheng-Prusoff equation [40] in which K_i _= (IC_50_)/(1+S), where S = [concentration of radioligand]/[K_D _of radioligand]. Comparisons between groups were made by two-way Student's *t*-tests or ANOVA and Tukey's or Dunnett's post hoc test (where appropriate; GraphPad Software, San Diego, CA), and groups were considered significantly different if *p *≤ 0.05.

## Abbreviations

IBMX, 3-isobutyl-1-methylxanthine; HBSS, Hank's balanced salt solution; UK 14,304, 5-Bromo-N-(4,5-dihydro-1H-imidazole-2-yl)-6-quinoxalinamine; ARC-239, 2-(2,4-(O-methoxyphenyl)-piperazin-1-yl)-ethyl-4,4dimethyl-1,3-(2H,4H)-isoquinolindione, AR, adrenoceptor; ISO, isoproterenol; EPI, epinephrine; NE, norepinephrine; wt, wild-type.

## Authors' contributions

TB-K participated in the design of the study, generated and selected stable SHβ_2_AR4-expressing clones, carried out all chronic treatment experiments, performed the statistical analyses and immunoblotting experiments, and drafted the manuscript. GFA carried out the binding and functional assays characterizing the α_2_-AR subtype. CDM conducted the molecular analysis studies. DCE participated in the conception and design of the study and helped draft the manuscript. LAS participated in the design and coordination of the molecular studies. KMS conceived of the study, participated in the design and coordination of all experiments and helped draft the manuscript. All authors read and approved the final manuscript.
